# Improved detection of circulating tumor cells in non-metastatic high-risk prostate cancer patients

**DOI:** 10.1038/srep39736

**Published:** 2016-12-21

**Authors:** Andra Kuske, Tobias M. Gorges, Pierre Tennstedt, Anne-Kathrin Tiebel, Raisa Pompe, Felix Preißer, Sandra Prues, Martine Mazel, Athina Markou, Evi Lianidou, Sven Peine, Catherine Alix-Panabières, Sabine Riethdorf, Burkhard Beyer, Thorsten Schlomm, Klaus Pantel

**Affiliations:** 1 Department of Tumor Biology, University Medical Center Hamburg-Eppendorf, Hamburg, Germany; 2 Martini Klinik, University Medical Center Hamburg-Eppendorf, Hamburg, Germany; 3 Laboratory of Rare Human Circulating Cells, University Medical Center, Montpellier, France; 4 Analysis of Circulating Tumor Cells Lab, Department of Chemistry, University of Athens, Athens, Greece; 5 Department of Transfusion Medicine, University Hospital Hamburg-Eppendorf, Hamburg, Germany

## Abstract

The relevance of blood-based assays to monitor minimal residual disease (MRD) in non-metastatic prostate cancer (PCa) remains unclear. Proving that clinically relevant circulating tumor cells (CTCs) can be detected with available technologies could address this. This study aimed to improve CTC detection in non-metastatic PCa patients by combining three independent CTC assays: the CellSearch system, an *in vivo* CellCollector and the EPISPOT. Peripheral blood samples from high-risk PCa patients were screened for CTCs before and three months after radical prostatectomy (RP). Combining the results of both time points, CTCs were detected in 37%, 54.9% and 58.7% of patients using CellSearch, CellCollector and EPISPOT, respectively. The cumulative positivity rate of the three CTC assays was 81.3% (87/107) with 21.5% (23/107) of patients harboring ≥5 CTCs/7.5 ml blood. Matched pair analysis of 30 blood samples taken before and after surgery indicated a significant decrease in CTCs captured by the CellCollector from 66% before RP to 34% after therapy (p = 0.031). CTC detection by EPISPOT before RP significantly correlated with PSA serum values (p < 0.0001) and clinical tumor stage (p = 0.04), while the other assays showed no significant correlations. In conclusion, CTC-based liquid biopsies have the potential to monitor MRD in patients with non-metastatic prostate cancer.

Prostate cancer (PCa) is the second most common cause of cancer-related death among men^1^. About 15% of patients with PCa are diagnosed with high-risk disease^2^. High-risk PCa, as defined by D’Amico (PSA ≥ 20 ng/ml AND/OR biopsy Gleason score ≥8 AND/OR clinical tumor stage ≥2c)^3^, includes a heterogeneous group of patients, some of whom display a lethal phenotype while others can be cured by treating the primary tumor alone. However, the optimal treatment management of these patients remains a significant challenge. Using the standard diagnostic procedures and radical prostatectomy treatment, up to 40% of patients are susceptible to biochemical disease recurrence (BCR) and 9% develop metastatic disease within 5 years^4^. Thus, radical prostatectomy must be considered in the context of multimodality treatment and the risk of (micro-) metastatic disease. To date, prostate-specific antigen (PSA) is the only approved biomarker guiding treatment decisions in PCa. However, PSA values often do not represent the current tumor status potentially leading to misinformed therapeutic decisions^5^. Furthermore, this biomarker only displays low predictive value in localized high-risk PCa patients and is unable to distinguish between pathologic stages. Thus, reliable surrogate markers that allow for the detection of minimal systemic disease and help guide the selective application of secondary therapies are urgently required.

Circulating tumor cells (CTCs) are promising biomarkers that have already shown prognostic relevance in metastatic breast, prostate, and colorectal cancer^6^,^7^,^8^. Furthermore, CTC counts were found to be superior to PSA in predicting overall survival (OS) in metastatic PCa (mPCa) and subsequently may be promising surrogate markers for therapeutic decision-making. Other interesting findings include CTC expression of androgen receptor splice variant-7 (ARv7) in predicting the efficacy of hormonal therapy^9^ and the discovery by Goldkorn and colleagues that CTC telomerase activity can also be a prognostic biomarker of OS^10^. In contrast, the clinical relevance of CTCs in non-metastatic PCa remains unclear. Even in high-risk PCa patients no correlation between clinicopathologic parameters (such as PSA value, Gleason grade or tumor stage) and CTC counts could be found, which may be due to low detection rates. According to published data, CTC positivity rates vary from 5–27% with an average of approximately 17%^11^,^12^,^13^,^14^,^15^. In light of this, it remains unclear if the available technologies are simply not sensitive enough for low level CTC detection or if patients with non-metastatic PCa have such low numbers of CTCs that they are not suitable candidates for CTC-based liquid biopsies. These are important questions to address for the biomarker field with important implications for future studies trying to detect therapeutic targets or identify resistance mechanisms in earlier stages of PCa. Using repeated prostate biopsies de Bono and colleagues recently demonstrated ARv7 expression already in hormone-sensitive PCa patients^16^.

The aim of this study was to increase the sensitivity of CTC detection in patients with high-risk PCa through the combination of three complementary assays: (i) the epithelial cell adhesion molecule (EpCAM)-dependent CellSearch technology, which received FDA-approval for metastatic prostate cancer, (ii) the GILUPI CellCollector (CellCollector) that captures EpCAM-positive CTCs *in vivo* by an antibody-coated needle introduced in the arm vein^17^,^18^,^19^, and (iii) the EpCAM-independent EPISPOT assay that enriches CTCs by negative depletion of leukocytes and detects viable prostate cancer cells based on their active secretion of PSA^20^. Peripheral blood was analyzed directly before and 3 months after radical prostatectomy (RP) to assess early dynamic changes in CTC counts, and the results were correlated to established risk factors.

## Results

### Patient’s characteristics

Eighty six and 52 patients were recruited for the first visit before RP and for the second visit 3 month after surgery, respectively. The clinical characteristics of the patients before RP are summarized in Table 1. The median age was 67 years, the median PSA concentration was 11.9 ng/ml, and the median Gleason score was 8.2. In total 84 of 86 patients underwent RP and eight patients additionally underwent hormone therapy. One patient was positive for a lymph node metastasis. During surgery lymph node metastases could be detected in 33 of 84 patients (39.3%).

### Comparison of CTC detection rates using different technologies

**Figure 1:**
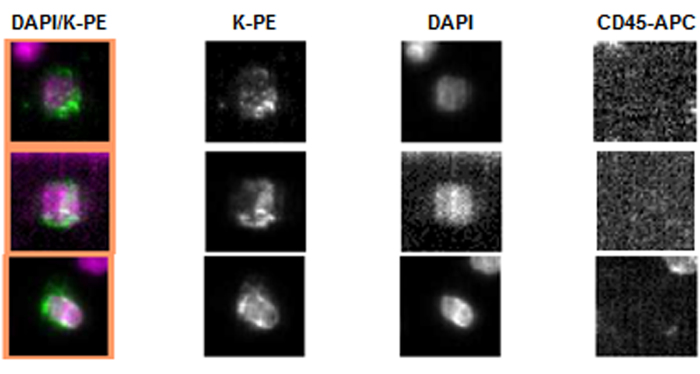
Representative pan-keratin positive (K-PE) and CD45 negative CTCs of high-risk PCa patients detected by the CellSearch System. DAPI was used for nuclear counter staining.

**Figure 2:**
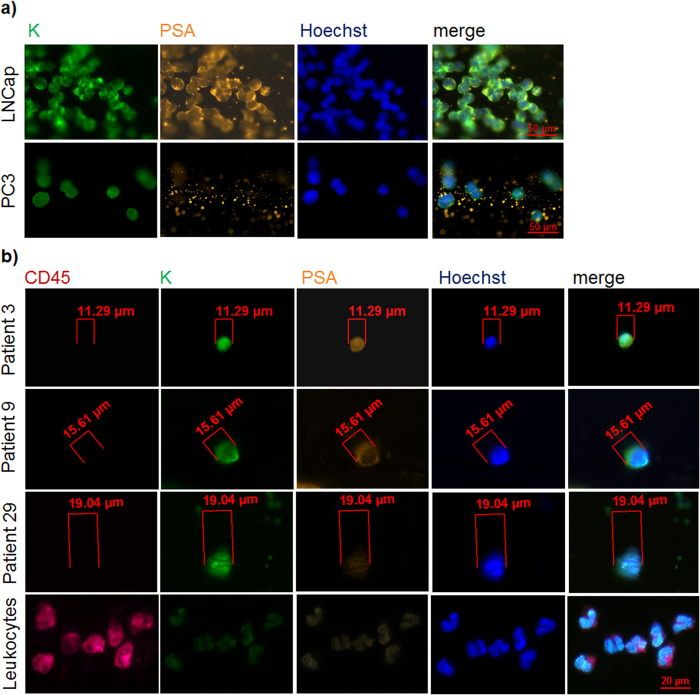
Immunofluorescence staining of established tumor cell lines or CTCs captured by the CellCollector. (**a**) Representative image of different prostate cancer cell lines isolated by the CellCollector in spiking experiments (*in vitro*). Tumor cells were identified as pan-keratin (K) positive events (green) and PSA positive (orange) (LNCap) or PSA negative (PC3) cell culture cells. (**b**) Representative CTCs isolated by the CellCollector (*in vivo*) and non-specifically bound leukocytes. Tumor cells were identified as pan-keratin positive (green), CD45 negative (red) and optional PSA positive (orange) events. Hoechst33342 (blue) was used for nuclear counterstain (Zeiss, Axiovision, 200× magnification).

**Figure 3:**
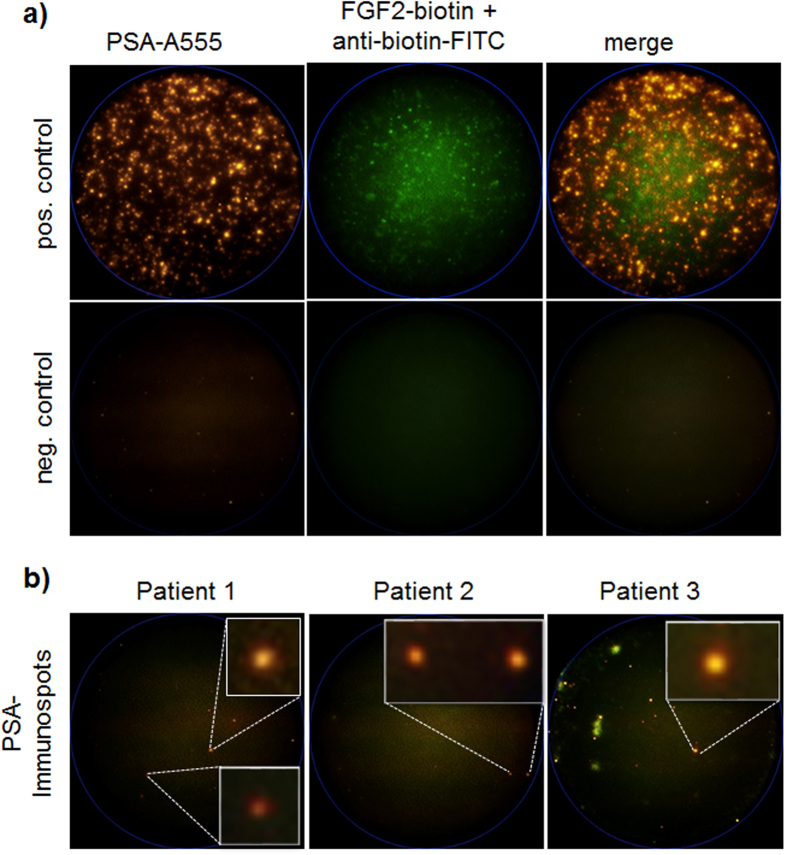
Representative example of PSA (orange) and FGF2 (green) immunospots detected by the dual fluoro-EPISPOT assay. (**a**) The LNCap and NBTII cell lines were used as a positive control for PSA and FGF2 secretion, respectively (positive control 2000 cells/cell line; negative control no cells). (**b**) PSA immunospots represent PSA-secreting cells defined as CTCs in prostate cancer (C.T.L. Elispot Reader, 50× magnification).

The CellSearch system was used as a “gold standard” in CTC detection in advanced prostate cancer^5^. Representative images of pan-keratin positive and CD45 negative CTCs detected by the CellSearch are displayed in Fig. 1. In addition to the CellSearch system, we applied the CellCollector as a novel approach to capture CTCs *in vivo*^17^. CTCs were identified as pan-keratin positive (green), leukocyte marker CD45 negative (red) and optional PSA positive (orange) events. Representative images of positive events (LNCap and PC3 cells as well as CTCs) or negative events (leukocytes) are displayed in Fig. 2. In total, 155 pan-keratin positive CTCs could be detected in 113 CellCollector samples, of which 71% (110/155) additionally expressed PSA. As a third technology, we used the EPISPOT assay, which is based on an EpCAM-independent enrichment method (i.e., leukocyte depletion) and enables the identification of viable PSA-secreting tumor cells. Figure 3a presents results of the proof of principal experiment in which LNCap and NBTII cell lines were used as a positive control for PSA and FGF2 secretion, respectively. Figure 3b displays PSA immunospots representing protein fingerprints of single viable PSA-secreting cells defined as functional CTCs obtained from the blood of prostate cancer patients. None of these CTCs secreted PSA and FGF2 simultaneously.

**Figure 4:**
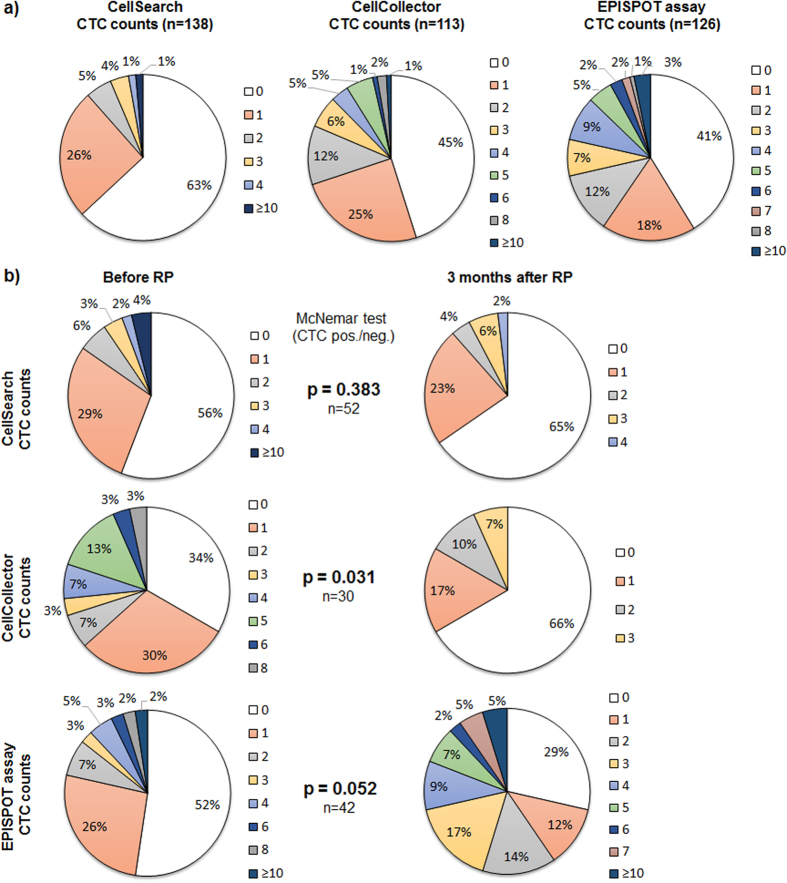
Distribution of detected CTCs applying the (i) CellSearch system (ii) CellCollector or (iii) EPISPOT assay. (**a**) Samples from both collection time points, i.e., one day before and 3 months after RP. (**b**) Distribution of detected CTCs in matched pairs applying (i) the CellSearch system (ii) the CellCollector or (iii) the EPISPOT assay before and three months after PRP. For statistical analysis the McNemar’s test was performed (p < 0.05 statistically significant).

**Figure 5:**
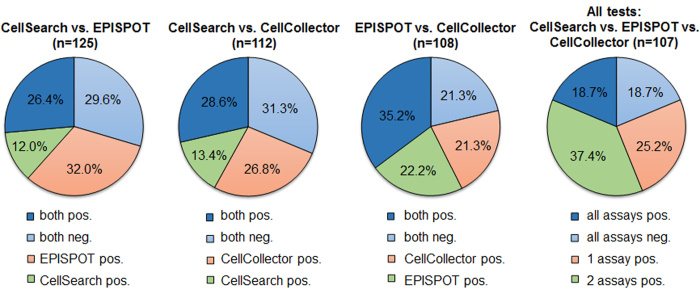
Concordance between two or all CTC detection assays. Assays were deemed to be concordant if the results were consistent, meaning both or all assays were either positive or negative for the same sample (blue). Assays were not concordant if the results were mismatched, meaning one assay was positive and the other negative (orange/green).

Combined application of the different CTC detection technologies revealed an improved detection rate of CTCs in high-risk PCa patients (Fig. 4a). Combining both collection time points, ≥1 CTCs were detected in 37–59% depending on the assay (CellSearch: 37% (51/138, range 1–10, median 1.8); CellCollector: 54.9% (62/113, range 1–12, median 2.4), and EPISPOT: 58.7% (74/126, range 1–13, median 3). The cumulative positivity rate (i.e., CTCs detected in at least one of the assays) was 81.3% (87/107), with 21.5% (23/107) of patients harboring >5 CTCs per blood sample. In total, only 18.7% of patients were positive for CTCs in all assays (20/107). The concordance between all three methods (all positive or all negative) was 37.4% and the paired concordance between two methods was similar, ranging from 56–60% (Fig. 5).

### Analysis of matched blood samples before and after surgery

Matched pair analysis of samples before and after surgery indicated a significant decrease of CTCs detected after treatment when using the CellCollector (Fig. 4b) (McNemar’s test p = 0.031 66% (range 1–8 CTCs, median 2.9) to 34% (range 1–3 CTC, median 1.7)). This significant difference was not observed with the CellSearch system (p = 0.383), while the EPISPOT assay showed a trend towards a higher CTC detection rate after RP (p = 0.052).

### Correlation of CTC incidence to clinical parameters

CTCs detected before RP by the EPISPOT assay significantly correlated to PSA serum concentrations and clinical tumor stage (Table 1). Before RP, patients who were negative for CTCs displayed a statistically significant lower PSA value in comparison to CTC-positive patients (p < 0.0001) (CTC neg.: Median 7.7 ng/ml (IQR 5.0–15.3); CTC pos.: Median 16.4 ng/ml, (IQR 8.3–40.4)). In addition, patients who were positive for CTCs identified by the EPISPOT assay had a more advanced tumor stage (p = 0.04). In contrast, no significant correlations could be obtained between any of these parameters and CTC counts detected by the CellSearch or CellCollector (PSA-CellSearch p = 0.16; PSA-CellCollector p = 0.62; tumor stage-CellSearch p = 0.24; tumor stage-CellCollector p=0.55) (Table 1). The parameter “Age” correlated statistically significant with CTC incidence detected by the CellCollector (p = 0.017) (CTC neg.: Median 68 (IQR 50–76); CTC pos.: Median 65 (IQR 44–75)).

## Discussion

In this study, the main objective was to assess the feasibility of detecting CTCs in non-metastatic high-risk PCa patients by combining the CellSearch platform, the *in vivo* CellCollector capture system and the EPISPOT assay. Our findings revealed, for the first time, an unexpectedly high incidence of CTCs. Published reports using the CellSearch system showed positivity rates of 5–27%^11^,^12^,^13^,^14^,^15^. Combining the results of both collection time points, we detected CTCs in 37%, 54.9%, and 58.7% of patients using CellSearch, the CellCollector and the EPISPOT, respectively. The cumulative positivity rate of all three CTC assays was 81.3% (87/107) with 21.5 (23/107) of patients harboring >5 CTCs per blood sample. In only 18.7% of patients, CTCs were found in all assays (20/107). This highlights the complementarity of these technologies. The advantage of combining EpCAM-dependent and EpCAM-independent technologies is based on the fact that an epithelial-to-mesenchymal transition (EMT) in CTCs can lead to a down-regulated EpCAM expression. Thus, EpCAM-based enrichment alone cannot detect all CTC subpopulations^21^. Moreover, the usual restriction of analyzing large blood volumes from cancer patients^22^ could be circumvented by the *in vivo* CellCollector capture system^17^,^18^, which resulted in higher incidences of CTC detection as compared to the CellSearch system. However, the number of isolated CTCs per sample was not significantly higher than obtained with the CellSearch system (CellSearch range 1–10, median 1.8; CellCollector range 1–12, median 2.4). Just recently, Theil and colleagues found a correlation between the number of CTCs captured by the CellCollector (cut off ≥5 CTCs) and overall survival^18^. However, this finding needs to be validated by additional and larger studies. To expand the range of the CellCollector or other *in vivo* capture technologies, additional antibodies against cell surface markers or EMT-associated targets expressed on/in CTCs such as N-cadherin, cell-surface vimentin or plastin 3 could be included^23^,^24^,^25^,^26^.

The paired analysis of patients before and after RP showed that CTC rates detected by the CellCollector displayed a statistically significant decrease after surgery (66% to 34%; p = 0.031). This indicates that after the removal of the primary tumor fewer EpCAM-positive CTCs would circulate in the blood stream, which can be explained by the fact that the primary tumor, a major source of CTCs, had been removed. CTCs have a short half-life time in blood^27^ and CTCs observed 3 months after complete removal of the primary tumor are derived most likely from disseminated tumor cells (DTCs)^28^ or small micrometastases. In contrast, the EPISPOT assay as the only EpCAM-independent technology, revealed a trend towards an increased amount of CTCs at 3 months after surgery (48% to 71%, p = 0.052). Whether this indicates the presence of EpCAM-negative DTCs/micrometastases, potentially associated with an increased risk of metastatic relapse, remains to be determined in future studies. In order to address this, a longer follow up time would need to be taken into consideration. Analysis of tissues from primary tumors and metastases of PCa patients revealed that only 11–13% of patients presented with EpCAM-negative or EpCAM-low tumors and the subset of EpCAM-negative tumor cells in these tumors was even lower^29^,^30^. However, EpCAM expression on CTCs in early stage PCa could be downregulated as the result of a selection process.

Detection of ≥1 CTCs by the EPISPOT assay before RP significantly correlated with clinicopathologic parameters such as PSA serum values (p < 0.0001) and clinical tumor stage (p = 0.04). CTC counts ≥5 did not correlate with any clinicopathologic parameter (e.g. gleason grade, tumor stage, PSA value). The correlation to PSA serum concentration is an interesting finding as the EPISPOT technology is based on viable PSA-secreting cells. The number of PSA-positive CTCs was also linked to the total tumor burden, as reflected by the tumor stage, which might be the most likely explanation for the correlation between CTCs and PSA serum levels. The extent to which PSA-positive CTCs directly contribute to PSA blood serum levels needs to be determined, however, this contribution should become more important at later stages when the primary tumor has been removed. Interestingly, co-secretion of FGF2, a potential stem cell growth factor, on PSA-secreting CTCs observed in a previous study that included PCA patients at more advanced stages^20^, was not found in this study, suggesting that CTCs in non-metastatic PCa patients might differ phenotypically from CTCs in advanced stage patients.

Although follow-up evaluations are now required to assess which CTC assay provides independent prognostic information, the present investigation opens a new avenue for investigations on minimal residual disease (MRD) in high-risk PCa patients due to the sufficient capturing of CTCs for enumeration and subsequent molecular characterization. Recent studies on tissue biopsies showed that mechanisms of resistance to anti-androgen therapies, such as the expression of Arv7, already exist in hormone-sensitive patients^16^. Early blood-based assessment of MRD will now allow sequential analyses of the evolution of MRD to overt metastases in PCa patients.

## Conclusion

Combining three complementary CTC assays has revealed, for the first time, a high incidence of CTCs in non-metastatic high-risk PCa patients. The observed correlation with established risk factors and the persistence of CTCs 3 months after surgery suggests a potential clinical relevance of CTCs as markers of MRD in PCa.

## Material and Methods

### Cell culture

Cell lines were obtained from the American Type Culture Collection: LNCaP (ATCC^®^ CRL-1740™), PC3 (ATCC^®^ CRL-1435™), NBTII (ATCC^®^ CRL-1655™). Cells were grown in a 75 cm^2^ flask until they reached a confluence of approx. 80%. Cells were trypsinized (Trypsin/EDTA (0.25% v/v, Gibco)), washed with sterile PBS (Gibco), and used for spiking experiments applying the CellCollector or the EPISPOT assay.

### Patients and blood collection

Between April 2014 and March 2016, 86 high-risk PCa patients (defined after D’Amico criteria^3^) were recruited for the application of (i) the CellSearch system, (ii) the CellCollector, and (iii) the EPISPOT assay. Eighty-four patients were treated with primary radical prostatectomy in the Martini-Klinik, Prostate Cancer Center at the University Medical Center Hamburg-Eppendorf, Germany. Blood samples were taken and CellCollectors were applied for 30 minutes in the vein of the patients one day before therapy (first visit) and three months after (second visit: 52/86 patients).

Blood samples from healthy individuals for spiking experiments were collected at the Department of Transfusion Medicine, University Hospital Hamburg-Eppendorf, Germany.

The study was carried out in accordance with the World Medical Association Declaration of Helsinki and the guidelines for experimentation with humans by the Chambers of Physicians of the State of Hamburg (“Hamburger Ärztekammer”). The experimental protocol was approved (Approval No. PVN-3779) by the Ethics Committee of the Chambers of Physicians of the State of Hamburg (“Hamburger Ärztekammer”). All included subjects provided written informed consent for participation in this study and the publication of results.

### CTC enrichment and detection technologies

#### CellSearch Circulating Tumor Cell Kit

7.5 mL of venous blood were collected in *CellSave tubes* and processed using the *CellSearch Circulating Tumor Cell Kit* (Janssen Diagnostics). This semiautomatic system contains a ferrofluid-based capture reagent targeting all EpCAM-positive cells, which could be further identified as CTCs by a pan-keratin and nuclear staining^31^. The CD45 staining was used to exclude contaminating leukocytes.

#### The CellCollector

(*In vitro*) For the evaluation of the CellCollector, LNCaP and PC3 cells were spiked into EDTA blood from healthy volunteers and incubated with the wire on a rotator for 30 min. After washing (PBS) and fixation (100% Acetone, 10 min), captured cells were permeabilized (PBS/0.1% TritonX-100) and blocked (PBS/3% BSA). Next, tumor cells were stained against pan-keratins (pan-keratin-A488, clone C11, dilution 1:50 in PBS/3% BSA; pan-keratin, clone AE1/AE2, dilution 1:50 in PBS/3% BSA), PSA (anti-PSA^A555^ Ab, clone H117, dilution 1:80 in PBS/3% BSA; this antibody was given by Prof. Hans Lilja, Memorial Sloan Kettering Cancer Center, New-York). CD45-staining was included to identify non-specifically bound leukocytes (CD45-A47, clone MEM-28, dilution 1:25 in PBS/3% BSA). Nuclear counter staining was performed using Hoechst (Hoechst 33258). Cells counted as tumor cells displayed: intact morphology, positive nuclear staining, pan-keratin signals, negative for CD45, cell diameter ≥4 μm, (and optionally positive for PSA).

(*In vivo*) For the *in vivo* application, the CellCollector was inserted through a conventional cannula (32 mm) and incubated in the patient’s vein^17^. Afterwards, the CellCollector was processed as described above.

To quantify CTCs, the CellCollector was fixed by a specific device, which enables the evaluation of the functionalized surface in 30° steps using a fluorescence microscope (Zeiss, Axiovision, 200× magnification).

#### The PSA/FGF2-EPISPOT assay

In order to separate erythrocytes and leukocytes from CTCs, RosettSep^TM^ reagent was added to 13–15 mL of EDTA blood (20 μL/mL) and processed following the manufacturer’s instructions (RosetteSep^TM^ CTC Enrichment Cocktail Containing Anti-CD56, STEMCELL technologies). During the dual fluorescent PSA/FGF2-EPISPOT assay, harvested tumor cells were cultured for two days on a membrane coated with antibodies against PSA (5 ng/μl H-50; this antibody was given by Prof. Hans Lilja, Memorial Sloan Kettering Cancer Center, New-York) and FGF2 (10 ng/μl 500-M38, PeproTech). The antibodies should capture the secreted PSA/FGF2 proteins that are subsequently detected by secondary antibodies labeled with fluorochromes (5 ng/μl PSA-H117 labeled with AlexaFluor^555^; 0.9 ng/μl FGF2-biotin + anti-biotin-FITC). Single fluorescent PSA and FGF2 immunospots were counted under a fluorescent microscope using a video camera imaging and computer-assisted analysis (KS ELISPOT, Carl Zeiss Vision) as well as with the CTL Elispot reader.

### Statistical analysis

For statistical evaluation with clinical-pathological variables CTC counts were stratified as negative or positive, with positive meaning at least 1 positive CTC was detected. To test for significance between CTC positivity and clinical-pathological variables, the Wilcoxon-test was used for continuous coded variables and the chi-square (likelihood) test for categorical variables. To compare the positivity of CTC prior to radical prostatectomy and 3 months after the McNemar test was used. To assess the clinical risk of a PSA progression after radical prostatectomy patients with negative and positive CTC were compared using the chi-square (likelihood) test. All tests were two-sided and a p-value < 0.05 was considered to be statistically significant. Data was analyzed using RStudio (version 0.99.491), an integrated development environment for R (version 3.2.2).

## Additional Information

**How to cite this article**: Kuske, A. *et al*. Improved detection of circulating tumor cells in non-metastatic high-risk prostate cancer patients. *Sci. Rep.* **6**, 39736; doi: 10.1038/srep39736 (2016).

**Publisher's note:** Springer Nature remains neutral with regard to jurisdictional claims in published maps and institutional affiliations.

## Figures and Tables

**Table 1:** Patient characteristics and correlation with CTC counts in blood samples obtained before RP.

Parameter	overall	CellSearch System			CellCollector			EPISPOT Assay			Combined		
		negative	CTC positive	p value	negative	CTC positive	p value	negative	CTC positive	p value	negative	CTC positive	p value
Patients; n (%)	86	53 (61.6)	33 (38.4)		31 (37.3)	52 (62.7)		40 (47.1)	45 (52.9)		17 (19.8)	69 (80.2)	
Age, Median (IQR), Min-Max	67 (60; 71)	67 (60;71), 47–76	67 (60;72), 44–75	0.88	68 (64; 73), 50–76	65 (58; 70), 44–75	**0.017**	67 (60;71), 50–76	67 (59;71), 44–76	0.8	67 (61; 73), 50–76	67 (60; 71), 44–76	0.65
BMI, Median (IQR), Min–Max	26.3 (24.6; 28.6)	26.2 (24.3;28.2); 18.1–38.1	26.5 (24.7;30.6); 18.9–42.6	0.27	25.5 (23.9; 28.4), 22.2–38.1	26.5 (24.9; 28.4), 18.1–42.6	0.21	26.4 (24.7; 28.4), 22.5–42.6	26.2 (24.3; 29.0), 18.1–33.5	0.62	25.6 (23.9; 30.0), 22.4–38.1	26.4 (24.6; 28.4), 18.1–42.6	0.74
PSA Median (IQR), Min-Max	11.9 (6.3; 26.6)	14.6 (7.5; 27.5), 0.39–98.5	8.3 (5.1; 23.8), 0.14–146.4	0.16	10.5 (5.9; 26.2), 0.14–74.0	11.9 (6.4; 26.8), 1.6–146.4	0.62	7.7 (5.0; 15.3), 0.14–61.8	16.4 (8.3; 40.4), 3.3–146.4	**<0.0001**	7.8 (3.4; 15.6), 0.4–28.6	14.2 (6.5; 31.8), 0.2–146.4	**0.03**
Biopsy Gleason Score; n (%)													
3+3	0 (0)	0 (0)	0 (0)	0.55	0 (0)	0 (0)	0.89	0 (0)	0 (0)	0.42	0 (0)	0 (0)	0.21
3+4	9 (10.5)	7 (13.2)	2 (6.1)		4 (12.9)	5 (9.6)		4 (10)	5 (11.1)		3 (17.6)	6 (8.7)	
4+3	5 (5.8)	3 (5.7)	2 (6.1)		2 (6.5)	3 (5.8)		1 (2.5)	4 (8.9)		0 (0)	5 (7.2)	
≥4+4	72 (83.7)	43 (81.1)	29 (87.9)		25 (80.6)	44 (84.6)		35 (87.5)	36 (80)		14 (82.4)	58 (84.1)	
Clinical T stage; n (%)													
T1c	37 (43)	23 (43.4)	14 (42.4)	0.24	11 (35.5)	25 (48.1)	0.55	19 (47.5)	18 (40)	**0.04**	6 (35.3)	31 (44.9)	0.25
T2a	20 (23.3)	12 (22.6)	8 (24.2)		10 (32.3)	10 (19.2)		11 (27.5)	8 (17.8)		7 (41.2)	13 (18.8)	
T2b	20 (23.3)	15 (28.3)	5 (15.2)		7 (22.6)	11 (21.2)		7 (17.5)	13 (28.9)		3 (17.6)	17 (24.6)	
T2c	5 (5.8)	1 (1.9)	4 (12.1)		1 (3.2)	4 (7.7)		0 (0)	5 (11.1)		0 (0)	5 (7.2)	
T3a	4 (4.7)	2 (3.8)	2 (6.1)		2 (6.5)	2 (3.8)		3 (7.5)	1 (2.2)		1 (5.9)	3 (4.3)	
Clinical N status; n (%)													
N_0_	84 (98.8)	52 (98.1)	33 (100)	0.32	30 (96.8)	52 (100)	0.16	39 (97.5)	45 (100)	0.22	16 (94.1)	69 (100)	**0.07**
N_1_	1 (1.2)	1 (1.9)	0 (0)		1 (3.2)	0 (0)		1 (2.5)	0 (0)		1 (5.9)	0 (0)	
M-status		For statistical analysis the McNemar’s test was performed (p < 0.05 statistically significant)											
Mx	54 (63.5)												
M_0_	31 (36.5)												
Hormonal therapy; n (%)													
yes	8 (9.4)												
No	77 (90.6)												
